# Selenium Nanoparticles Control H1N1 Virus by Inhibiting Inflammatory Response and Cell Apoptosis

**DOI:** 10.3390/molecules28155920

**Published:** 2023-08-07

**Authors:** Jingyao Su, Jia Lai, Jiali Li, Chuqing Li, Xia Liu, Chenyang Wang, Bing Zhu, Yinghua Li

**Affiliations:** Center Laboratory, Guangzhou Women and Children’s Medical Center, Guangzhou Medical University, Guangzhou 510120, China; sujingyao@stu.gzhmu.edu.cn (J.S.); 2022210293@stu.gzhmu.edu.cn (J.L.); wcy@stu.gzhmu.edu.cn (C.W.);

**Keywords:** SeNPs, H1N1, inflammation, apoptosis

## Abstract

The treatment of influenza caused by H1N1 has been the focus of much attention. Selenium nanoparticles (SeNPs) have been used in many aspects of research in the last two decades. They have shown excellent performance in antiviral, anti-inflammatory, and antioxidant functions. Previous anti-H1N1 cell experiments using SeNPs have shown that they have evident antiviral effects and low toxicities. This study focuses on the mechanism of selenium nanoparticles against an H1N1 influenza virus infection in vivo. The results showed that the selenium levels in the body decreased after an H1N1 virus infection, and inflammatory factors in the lung tissues increased abnormally, leading to the onset and aggravation of an inflammatory response. The H1N1 virus infection also led to the excessive activation of apoptotic pathways in the body and induced the apoptosis of tissue cells. In addition, this study found that SeNPs can alleviate this phenomenon. All results showed that SeNPs are promising inhibitors for controlling influenza H1N1 virus infections.

## 1. Introduction

Influenza is a potentially serious respiratory infection caused by the influenza virus [1]. Most human cases are caused by influenza A virus strains of the subtypes H1N1 and H3N2 [2]. Influenza A virus (IAV) is the most common infectious agent in humans, infecting about 10–20% of the world’s population and causing 30,000–50,000 hospitalizations each year [3]. The current treatments for influenza are antiviral drugs and vaccination for prevention [4]. Immunization is a favorable, safe, and efficacious method of influenza prevention, but policies and vaccinations vary around the world [5]. Vaccination commonly experiences problems with operational challenges and controversial issues regarding its effectiveness and necessity, but there is a lack of approaches to overcome these barriers [6].

For the human body, selenium is an important trace element [7]. A selenium deficiency causes certain effects on thyroid [8] and cardiovascular functions [9] and may also increase the susceptibility to infection [10]. Therefore, correcting a selenium deficiency is a meaningful measure of health [11]. As nanoparticles reduce toxicity and improve targeting, they are used as a carrier to deliver various drugs to the site of action [12]. More accurate drug delivery characteristics have led to extensive experimentation. Selenium nanoparticles (SeNPs) have received extensive attention and have been the subject of much research in the last two decades [13]. Starting from an immune direction has become another way to study the treatment of some diseases. Some studies have shown that selenium nanoparticles play a role in the regulation of inflammation and are able to combat various diseases caused by chronic inflammation [14]. Rao et al. [15] loaded astragalus polysaccharide (APS) and tanshinone IIA (TSIIA), traditional active ingredients in Chinese medicine, on selenium nanoparticles (TSIIA@SeNPs-APS). It was found that TSIIA@SeNPs-APS could significantly improve the efficiency of the cellular uptake of the drug, and the drug worked better, which ultimately protected the spinal cord neurons of rats. There are studies on the use of selenium nanoparticles for the relief of inflammation in the intestinal tract [16]. Some are used to prevent cardiovascular disease [17], and some studies have investigated their use in fighting infectious diseases [18]. SeNPs hold promise for immunotherapy, such as against cancer and chronic diseases. In addition, SeNPs are more effective than traditional forms of selenium at synthesizing selenoproteins, helping the nutrients to be better absorbed [19]. The outstanding performance of selenium nanoparticles in various aspects makes us want to see their anti-H1N1 roles. 

During influenza virus infections, the body’s immune mechanisms are activated, and oxidative stress occurs. The excessive accumulation of cytokines and immune cells leads to a severe inflammatory response, lung disease, and a decline in lung function [20]. Common immune abnormalities can cause an increase in INF-α, interleukin (IL)-6, IL-1β, pro-inflammatory chemokines, activated macrophages, and the nuclear factor kappa beta (NF-κB) [21]. Activated NF-κB regulates the expression of inflammatory factors such as IL-4, IL-6, and IL-8 and triggers an inflammatory response [22]. In a study by Abood et al. [23], it was found that influenza infections increased the lung IL-22 production, and an IL-22BP deficiency reduced repeat influenza virus infections. Yuan et al. [24] found that highly pathogenic viral infections triggered abnormal increases in a large number of cytokines, especially IL-8, IL-17, and pro-inflammatory factors, causing complications and, eventually, death due to multi-organ failure. The influenza virus caused elevated NF-κB, IL-1β, TNF-α, and IL-6 levels, leading to severe lung tissue lesions in mice, which were alleviated by rhodopsin, as found by Dai et al. [25]. Zhu et al. [26] found that Jinhua Qinggan granules (JHQG) could alleviate lipopolysaccharide-induced acute lung injury (ALI) by inhibiting the expression of the anti-apoptotic proteins Bcl-XL and Mcl-1 and increasing the apoptosis of neutrophils. 

In a previous study, selenium nanoparticles were investigated at the cellular level and found to be resistant to the H1N1-mediated apoptosis of MDCK cells [27]. Unlike previous studies, this paper constructed a model of selenium-deficient mice by feeding them selenium-deficient diets and explored whether selenium nanoparticles, which were not loaded with drugs and had antiviral effects and mechanisms in selenium-deficient mice. SeNPs were used to experiment on H1N1-infected mice to explore the mechanisms of the changes in selenium content, inflammatory factors, and apoptotic proteins in tissues. This study aimed to provide a reference for research on the antiviral effects of selenium nanoparticles.

## 2. Results and Discussion

### 2.1. Drug Toxicity and Antiviral Results of SeNPs

**Figure 1:**
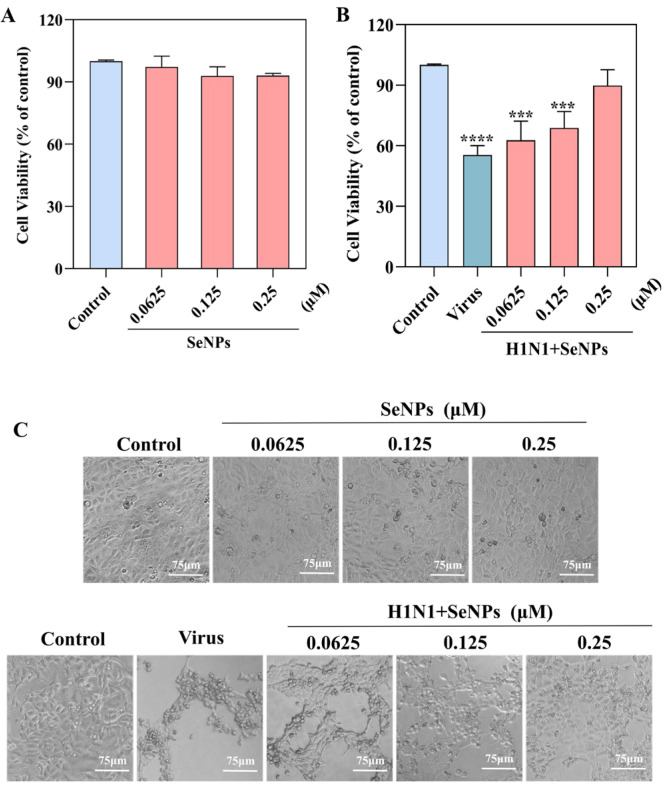
The effect of SeNPs on the growth of MDCK cells was determined by CCK8 assay. (**A**) A cytotoxicity assay to test whether different concentrations of SeNPs are toxic to MDCK. (**B**) Anti-viral assay: to test whether SeNPs can inhibit the replication of the H1N1 influenza virus. (**C**) Phase contrast microscopy to observe whether different concentrations of SeNPs could affect the morphology of MDCK cells and whether different concentrations of SeNPs could inhibit the cell damage caused by the H1N1 influenza virus. Bars with different characters are statistically different at *p* < 0.0001 (****) and *p* < 0.001 (***). The scale bar in the figure is 75 μm.

The drug toxicity and antiviral effects of SeNPs were assayed using cck-8. From the results shown in Figure 1A, it is clear that SeNPs were not toxic at 0.0625, 0.125, or 0.25 µM compared with controls. The antiviral effects of SeNPs are shown in Figure 1B; after treatment with SeNPs, there were concentration-dependent increases at 0.0625, 0.125, and 0.25 µM compared with the H1N1 group. Figure 1C shows that the cell morphologies of the MDCK cells infected with the H1N1 influenza virus changed from a pyknotic flattened wall state to a crumpled cluster of cells, resembling a grape-bunch shape. This was also the typical state of microscopic MDCK cells infected with the H1N1 influenza virus, but it could be seen that the cell morphologies gradually approached those of the control group after the addition of SeNPs, which also reflected that the SeNPs did effectively inhibit the cell damage caused by the H1N1 influenza virus infection. In the group with SeNPs alone, it was found that the cellular state was also similar to that of the control group, which reflected the advantage of the SeNPs having low toxic effects on the cells. The results above show that SeNPs had an anti-H1N1 effect, and the best effect was observed at 0.25 µM, which was consistent with the concentrations studied previously.

### 2.2. The Weight Change and Lung Inflammation in H1N1 Mice

**Figure 2:**
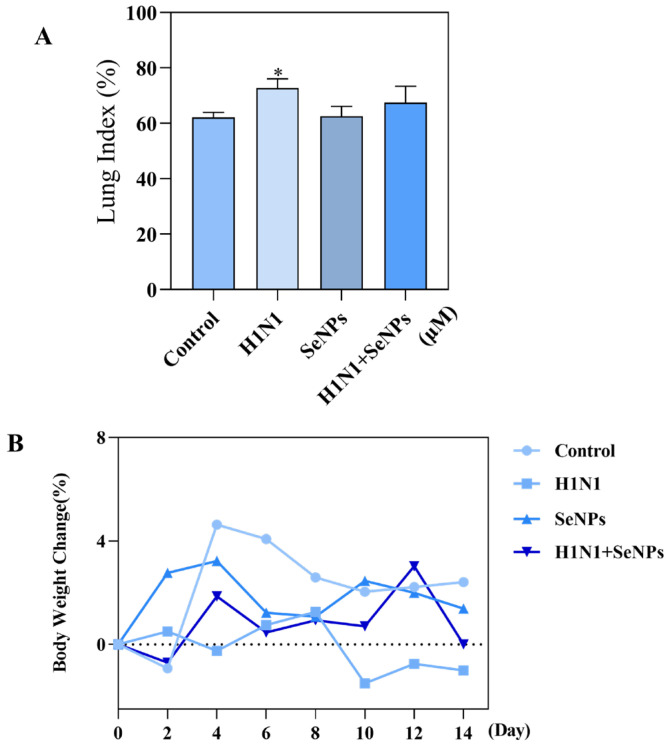
SeNPs inhibited the damage induced by H1N1 infection in selenium-deficient mice. (**A**) Mouse lung index: difference in lung index between selenium-deficient mice infected with H1N1 influenza virus and selenium-deficient mice after treatment with SeNPs. (**B**) Mouse body weight: Changes in body weight of selenium-deficient mice infected with the H1N1 influenza virus. Bars with different characters are statistically different at * *p* < 0.05.

To explore the role of SeNPs in mice infected with H1N1, we used nasal drops to infect mice with the H1N1 influenza virus, gavaged the mice with SeNPs for three consecutive days after infection with the virus, and, finally, sacrificed the mice on day 14 for follow-up experiments. Figure 2A demonstrates the weight change in mice. The weight of the mice kept decreasing after infection. In contrast, no body weight decreased in mice treated with SeNPs. The lung index was obtained by comparing lungs’ mass to the body’s mass and reflected the degree of pneumonia [28]. From the results of Figure 2B, it was clear that the lung index of mice was higher in the group infected with the H1N1 influenza virus compared to the control, whereas it approached that of the control group after drug treatment with SeNPs. The above results showed that H1N1 infection caused a pulmonary inflammatory response in mice, which should confirm that influenza virus infection may cause a cytokine storm and thus an inflammatory response in vivo [29]. The addition of SeNPs effectively alleviated the inflammatory exudation caused by the H1N1 influenza virus and reduced the symptoms of weight loss in mice after virus infection.

### 2.3. The Inflammatory Cytokine in H1N1 Mice

**Figure 3:**
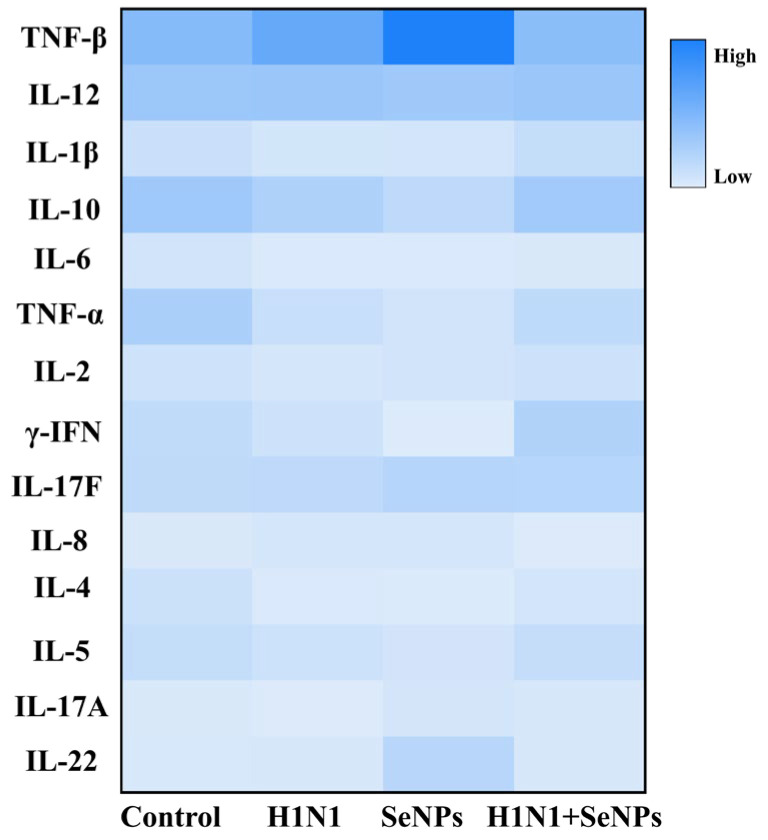
Regulation of inflammatory factors in vivo by SeNPs and alteration of corresponding cytokines in selenium-deficient mice infected with H1N1 influenza virus after treatment with SeNPs.

Cytokine levels can reflect the degree of inflammation. The results shown in Figure 3 show that influenza virus infection could significantly cause increases in TNF-β, IL-8, and IL-22, and the addition of SeNPs decreased TNF-β, IL-8, and IL-22. In addition, influenza virus infection also caused decreases in IL-1β, IL-2, IL-4, IL-6, IL-10, and IL-17F, and the SeNPs-only group was able to increase IL-6, IL-10, and IL-17F, but the therapeutic effect (H1N1 + SeNPs group) was not significant. The above analysis suggests that influenza-virus-mediated inflammatory response may be associated with the IL family, and SeNPs could inhibit inflammatory factors to suppress the inflammatory response.

### 2.4. Results of Apoptosis and Inflammatory Response in Mouse Lung Tissue

**Figure 4:**
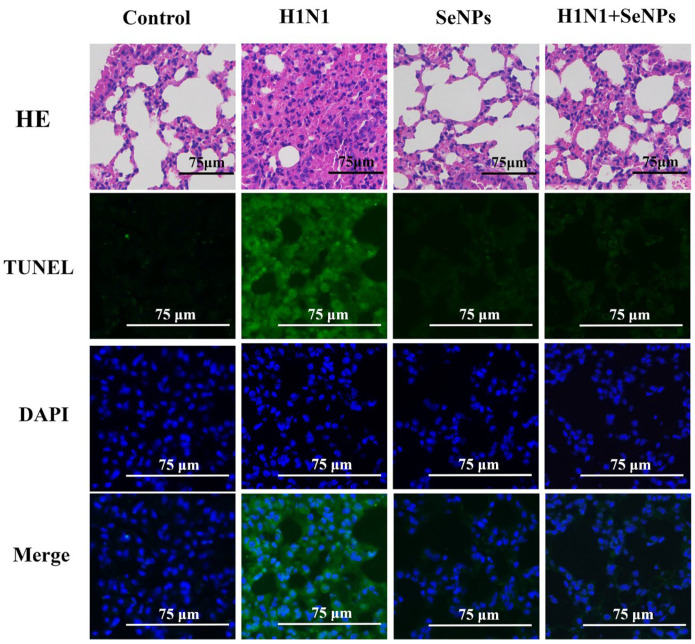
HE staining was used to observe the changes in lung morphology, inflammatory exudation, and apoptotic responses in mice infected with H1N1 influenza virus and treated with SeNPs in selenium-deficient mice. The scale bar in the figure is 75 μm.

The TUNEL-DAPI staining method could detect apoptotic cells, showing green fluorescence [30]. Figure 4 shows the most vigorous green fluorescence intensity in the H1N1 virus group by fluorescence microscopy. It meant that the cells of lung tissues showed extensive apoptosis after infection with the H1N1 influenza virus and a corresponding increase in nuclei breakage after infection with the influenza virus using DAPI staining. The results of the SeNPs group and the H1N1 + SeNPs group were similar to the control group. These results indicated that the drug SeNPs did not harm the cells, reflecting their low toxicities. The results also showed that the addition of SeNPs alleviated the occurrence of apoptosis and nucleus disruption caused by H1N1 influenza virus infection. In addition, the HE staining results also suggested that SeNPs inhibited the effect of influenza-virus-induced inflammatory response, as seen in HE-stained sections of lung tissue infected with H1N1 influenza virus, in which alveoli fused to form larger cavities, and inflammatory infiltration between tissues increased significantly. Still, the lung structure returned to a typical reticular structure after adding SeNPs, and lymphocytes also decreased accordingly. SeNPs effectively inhibited the apoptosis induced by H1N1 virus infection and the lung’s inflammatory response.

### 2.5. Immunohistochemical Detection of Mouse Lung Tissue

**Figure 5:**
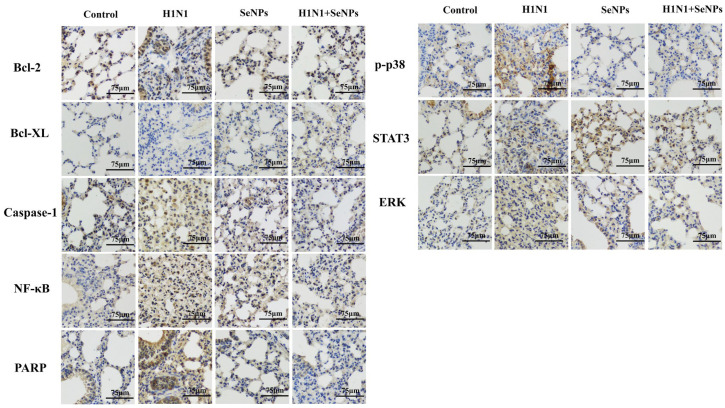
SeNPs inhibited H1N1-infection-mediated injury in selenium-deficient mice. Lung tissues from selenium-deficient mice with different administration methods were embedded and sectioned for immunohistochemical assays to detect the expression of inflammation and apoptosis-related proteins in the lung tissues of selenium-deficient mice. The scale bar in the figure is 75 μm.

The mice were grouped and administered in different ways. After 14 days of execution, lung tissue sections were removed and immunohistochemically stained for apoptosis and inflammation-related proteins [31]. As shown in Figure 5, the immunohistochemical results of Bcl-2, Bcl-XL, PARP, and other proteins suggested that the addition of SeNPs inhibited the development of apoptosis after influenza infection. The immunohistochemical results of STAT3, Casepase-1, p-p38, and other proteins suggested that SeNPs were likely to modulate the body’s immune response against the inflammatory response caused by H1N1 influenza virus infection. The results mentioned above all supported the verification of the occurrence of apoptosis and inflammatory response during H1N1 influenza virus infection. With the addition of SeNPs, the apoptosis status and inflammatory factor secretion were reduced to some extent, thus achieving the inhibition of influenza virus infection.

### 2.6. Selenium Levels in Tissues and Serum of Mice

**Figure 6:**
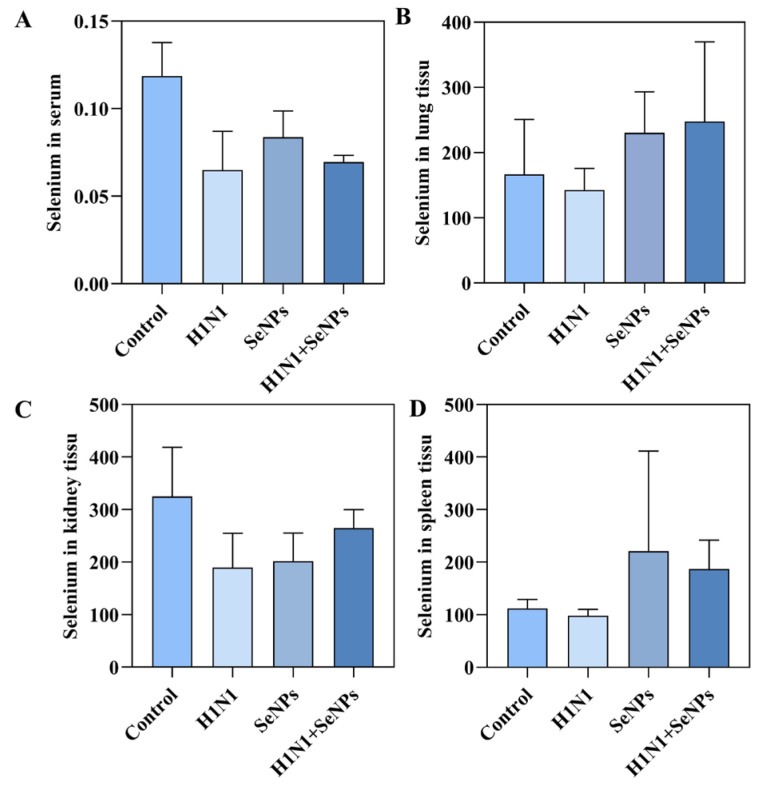
SeNPs modulate selenium content in mice to inhibit H1N1 influenza virus infection. (**A**) Total selenium in mouse serum was measured by ICP-MS. (**B**) Determination of total selenium in the lung tissue of mice by ICP-MS. (**C**) Determination of total selenium in the kidneys of mice by ICP-MS. (**D**) Determination of total selenium in mouse spleen by ICP-MS.

The selenium contents in mice organ tissues and serums were measured using atomic fluorescence spectrometry [32]. As shown in Figure 6, it can be found that the selenium contents in both mouse serum, lungs, spleen, and other tissues and organs decreased accordingly after infection with the H1N1 influenza virus. However, the selenium contents in tissues showed different degrees of increase after treatment with SeNPs. Selenium played an vital role in viral infections in previous studies [33], and this study used SeNPs to supplement the selenium content of the body to achieve regulation of the immune system and alleviate the inflammatory response caused by H1N1 influenza virus infection.

## 3. Discussion

The H1N1 virus is the most predominant subtype that causes influenza, mainly because of its high pathogenicity and infectiousness [1]. Seasonal flu is primarily transmitted by droplets but also indirectly through mucous membranes. It is generally acute with mild manifestations of fever, cough, and sputum, easily confused with the common cold, respiratory distress, or even death by failure in severe cases [34]. Treatment for H1N1 has also been a point of concern for researchers, and vaccination can play a role in preventing infection and painful illness, but the discovery of a drug that is effective in its treatment is of more significant concern. The drugs that have been used clinically to treat the M2 ion channel and neuraminic acid inhibitors have been used for many years unchanged. Still, the virus has been changing, and drug resistance has become common [35]. In this study, nanoparticles and selenium were combined to investigate whether selenium nanoparticles (SeNPs) could exert antiviral effects in mice. Therefore, studying the pathogenic mechanism of the H1N1 influenza virus is necessary. Discovering a drug that could treat the influenza virus is a must.

Previously, our team found that selenium nanoparticles exhibited low toxicities and antiviral effects in MDCK cell experiments and were able to inhibit influenza-virus-mediated apoptosis. Therefore, in this article, we constructed selenium-deficient mice and continued to explore whether selenium nanoparticles (SeNPs) could play an antiviral role in selenium-deficient mice. Firstly, it was found that SeNPs at 0.25 μM had low cytotoxicities and high antiviral effects by the CCK-8 experiment. In the in vivo test, a dose of 2.5 mM, a single injection of 20 μL, was chosen for the mouse experiment after searching the literature to set the concentration. In H1N1-infected mice for 14 days, the body weight of the mice continued to decrease, and the lung index of the mice increased significantly. The pulmonary index is an objective expression of the degree of pneumonia. The increase in the lung index of the infected mice indicated the presence of some degree of pneumonia in the mice. Therefore, the experiment was further tested for inflammatory factors in the serum of the mice.

Interleukin 22 (IL-22) is a member of the IL-10 cytokine family. It plays a vital role in antimicrobial defense, homeostasis, and tissue repair, and it has a dual role in inflammation in various diseases [36]. From the resulting graph demonstrated that influenza virus infection could increase in TNF-β, IL-8, and IL-22, and, after the intervention of SeNPs, TNF-β, IL-8, and IL-22 were significantly decreased. It has been shown that activated NF-κB can regulate IL-8 production and trigger inflammatory responses [37]. Combined with the immunohistochemical NF-κB results, H1N1 infection caused an increase in NF-κB in hypotheses, which stimulated a further rise in IL-8 and aggravated the inflammatory response caused by influenza. During infection, the influenza virus causes a cytokine storm, resulting in a series of inflammatory reactions [38]. The inflammatory response caused by the influenza virus results from multiple cytokines working together, and SeNPs can alleviate the inflammatory response caused by H1N1 by regulating cytokines. The HE staining results after infection with the H1N1 virus showed inflammatory cell infiltration and edema in lung tissue and fusion of alveoli in lung tissue, further indicating that a severe inflammatory response occurred in the lung tissue of mice after influenza virus infection. After the SeNPs treatment, the inflammatory factors and the lung tissues of mice were restored to normal, indicating that SeNPs could effectively inhibit the H1N1-mediated inflammatory response.

It has also been shown that inflammatory responses and apoptosis are induced during influenza virus infection [39]. Therefore, we performed TUNEL-DAPI staining to observe apoptosis and DNA damage. TUNEL was able to label the 3′-OH ends of the broken DNA strands of the apoptotic cells, producing green fluorescence, and the fluorescence plot showed that a large number of apoptotic cells occurred in the H1N1-infected lung tissue. DAPI could stain the nuclei and DNA blue, and the resultant plot showed that many DNA breaks occurred after the H1N1 infection, and the loops became fragmented. The green fluorescence was significantly reduced after adding SeNPs, and the fragmented blue fluorescence was reduced. After observing the phenomenon of apoptosis, immunohistochemistry was performed on some of the apoptotic proteins to further investigate the mechanism of apoptosis.

Immunohistochemical results after infection with the H1N1 virus showed a loss of alveolar structure and alveolar fusion in lung tissues, consistent with the HE staining results. The inflammatory response of the body after infection requires the initiation of the NF-κB signaling pathway, and the inflammatory factors TNF-α and IL-1β activate NF-κB, which regulates the production of the Bcl-2 anti-apoptotic protein [40,41]. It is known that P38 is one of the classical proteins that regulate apoptosis [42]. STAT3 can be activated by interleukins, such as IL-4,6,10, and 12, STAT3 can promote or inhibit Bcl-XL, and the disruption of STAT can directly lead to apoptosis and inflammatory response [43,44,45]. The above results illustrate suggests that inflammatory factors and apoptotic proteins are mutually promoting during influenza virus infection; selenium nanoparticles can regulate this process.

On the other hand, it has been shown that selenium contributes to the composition of the active part of enzymes and participates in the immune cycle [46]. Therefore, in this article, lung, kidney, and spleen tissues and mice serums were also taken to determine selenium contents. It was found that the selenium contents in the tissues and serums of the mice infected with H1N1 were significantly reduced, indicating that influenza virus infection led to a deficiency of selenium content. In contrast, the addition of the drug resulted in a significant increase in selenium content in tissues, especially lung tissues. A description of all the results suggested that SeNPs could replenish selenium levels, which may play an antiviral role to some extent.

## 4. Materials and Methods

### 4.1. Materials

MDCK cells were obtained from ATCC CCL-34TM. H1N1 influenza virus was obtained from Guangzhou Women and Children Medical Center, Guangzhou Medical University. CCK-8 kits were purchased from Biotime Biotechnology. A TUNEL-DAPI co-staining kit was purchased from Sigma-Aldrich (Darmstadt, Germany). Bcl-2, Bcl-XL, Caspase-1, NF-κB, PARP, p-p38, STAT3, and ERK antibodies were purchased from Cell Signaling Technology (Danvers, MA, USA).

### 4.2. Antiviral Effects Test

The cytotoxic and antiviral effects of SeNPs were tested by CCK-8 assay [47]. MDCK cells were cultured in 96-well plates and grown for 24 h, followed by H1N1 infection for 2 h and additions of 0.0625, 0.125, and 0.25 µM SeNPs for 48 h. Then, we added CCK-8 reagent, incubated at 33 ℃ for 1 h, and detected the absorbance at 540 nm by enzyme markers. The cytotoxic and antiviral effects of SeNPs were assessed by cell viability. Experiments were repeated 3 times (n = 3).

### 4.3. The Body Weight and the Lung Index Calculated

To investigate whether H1N1 affects body weight and lung function in mice. We prepared four groups of female C57 mice, 24 in total, from 4–6 weeks of age. Construction of a selenium-deficient mouse model was performed by feeding them with selenium-deficient chow. The groups were set up as the control, H1N1, SeNPs, and H1N1 + SeNPs groups. The mice were first anesthetized with 10% chloral hydrate at a dose of 3 μL/g. Then, the H1N1 group and the H1N1 + SeNPs group were treated with 20 μL of H1N1 dilution in a nasal drip at a dose of 1000 TCID50/0.1 mL. The control group was treated with 20 μL of saline in a nasal drip. After 24 h, 2.5 mM of SeNPs was added to mice in the SeNPs group and the H1N1 + SeNPs group, and nasal drops were performed for three consecutive days. It was necessary to record the mouse’s weight daily and to observe its condition. After 14 days, mice were anesthetized and executed after weighing, and eye blood and lung tissues were taken. Lung tissues were weighed for calculation of lung index.

### 4.4. Detection of Cytokines 

A total of 20 μL of the bead mixture, 20 μL of the sample solution, and 20 µL of testing antibodies were added to a 96-well filter plate and then incubated at 500 rpm for 2 h in a black environment. The second step was putting 20 μL of PE-labeled Streptomyces-plant cyanate (SA-PE) solution into the wells for 30 min with 500 rpm at room temperature [48]. After obtaining samples, flow cytometry was used to detect fluorescence in the samples. In the last step, the fluorescence was obtained with a fluorescence cytometer, and the level of cytokines in the sample could be obtained by calculating the fluorescence intensity. The software used was BD FACS Diva software v8.0.3 (collection and analysis of fluorescence data) [49].

### 4.5. TUNEL-DAPI Co-Staining, HE Staining, and Immunohistochemistry

After 14 days, the cervical vertebra of mice were dislocated, and lung tissues were taken and fixed in paraformaldehyde. Lung tissues were fixed, dehydrated, embedded in wax, sectioned, and then stained with TUNEL-DAPI and HE, respectively. Detection of apoptotic cells was performed using the TUNEL-DAPI Apoptosis Kit [30]. DAPI is a cell nuclear dye. During apoptosis, the nuclear DNA breaks, so DNA breaks could be observed by fluorescence microscopy to assess the level of apoptosis. Hematoxylin-eosin (HE) could make the nuclei blue as a way to see the distribution of cells in the lung tissue. For immunohistochemistry, after quenching with 3% H_2_O_2_ and blocking, the slices were incubated with Bcl-2, Bcl-XL, STAT3, NF-κB, Caspase-1, PARP, p-p38, and ERK at 4 °C overnight [31].

### 4.6. Determination of Selenium Content in Lung Tissue and Serum of Mice

First, 0.5 g of mouse lung tissue and 100 μ of serum were taken, mixed with 1 mL of perchloric acid and 3 mL of nitric acid, and digested overnight. Then, we heated and cooled 3 mL of hydrochloric acid and continued heating until the solution became clear. Finally, the selenium content in mouse lung tissue and serum was measured by atomic fluorescence spectrometry (AFS) [32].

### 4.7. Statistical Analysis

All data are presented as mean ± standard deviation. One-way analysis of variance (ANOVA) was used for comparison between multiple groups. *p* < 0.05 (*) or *p* < 0.01 (**) was considered a statistically significant difference.

## 5. Conclusions

In this study, we refined the anti-H1N1 effects of selenium nanoparticles (SeNPs) in vitro and in vivo and further explored their antiviral mechanisms from the changes in lung tissues, cytokines, and selenium contents in tissues and serums of mice. The results showed that H1N1 virus infection resulted in a decrease in selenium levels and an abnormal increase in inflammatory factors in lung tissue, leading to the onset and aggravation of the inflammatory response. H1N1 infection also led to excessive activation of apoptotic pathways, leading to apoptosis in tissue cells. The combined effect eventually led to a loss of appetite and significant weight loss in mice. SeNPs could act as anti-H1N1 by reducing the inflammatory response and apoptosis through supplementing selenium levels or directly inhibiting the production of inflammatory factors and apoptotic proteins. However, the mechanisms of SeNPs against H1N1 influenza, as well as their mediated apoptoses and inflammatory responses, still need to be investigated. The selenium-deficient mouse model constructed in this paper needs to be further explored to supplementing selenium levels to combat influenza mechanisms. The above results suggested that selenium nanoparticles are promising and worthy of further investigation as an antiviral drug.

## Data Availability

Data sharing does not apply to this article as no datasets were generated or analyzed during the current study.
